# Accelerometer-Based Step Regularity Is Lower in Older Adults with Bilateral Knee Osteoarthritis

**DOI:** 10.3389/fnhum.2016.00625

**Published:** 2016-12-08

**Authors:** John M. Barden, Christian A. Clermont, Dylan Kobsar, Olivier Beauchet

**Affiliations:** ^1^Faculty of Kinesiology and Health Studies, Neuromechanical Research Centre, University of ReginaRegina, SK, Canada; ^2^Faculty of Kinesiology, University of CalgaryCalgary, AB, Canada; ^3^Division of Geriatric Medicine, Department of Medicine, McGill UniversityMontreal, QC, Canada

**Keywords:** knee osteoarthritis, accelerometry, gait regularity, gait symmetry

## Abstract

**Purpose:** To compare the regularity and symmetry of gait between a cohort of older adults with bilateral knee osteoarthritis (OA) and an age and sex-matched control group of older adults with healthy knees.

**Methods:** Fifteen (8 females) older adults with knee OA (64.7 ± 6.7 years) and fifteen (8 females) pain-free controls (66.1 ± 10.0 years) completed a 9-min. walk at a self-selected, comfortable speed while wearing a single waist-mounted tri-axial accelerometer. The following gait parameters were compared between the two groups according to sex: mean step time, mean stride time, stride and step regularity (defined as the consistency of the stride-to-stride or step-to-step pattern) and the symmetry of gait (defined as the difference between step and stride regularity) as determined by an unbiased autocorrelation procedure that analyzed the pattern of acceleration in the vertical, mediolateral and anteroposterior directions.

**Results:** Older adults with knee OA displayed significantly less step regularity in the vertical (*p* < 0.05) and anteroposterior (*p* < 0.05) directions than controls. Females with knee OA were also found to have significantly less mediolateral step regularity than female controls (*p* < 0.05), whereas no difference was found between males.

**Conclusion:** The results showed that the regularity of the step pattern in individuals with bilateral knee OA was less consistent compared to similarly-aged older adults with healthy knees. The findings suggest that future studies should investigate the relationship between step regularity, sex and movement direction as well as the application of these methods to the clinical assessment of knee OA.

## Introduction

The ability to locomote is a fundamental activity for all humans, and is an essential component of maintaining independence and a healthy quality of life. However, it is understood that the physiological changes that accompany aging in the last decades of life increasingly challenge the neuromuscular system to maintain consistent levels of mobility. Often these changes are exacerbated by other disease mechanisms or processes in one or more systems, to the extent that gait function can be severely compromised. Osteoarthritis (OA) is a debilitating disease that involves the progressive degradation of articular cartilage in the body's major weight bearing joints (Baliunas et al., 2002). It affects millions of people worldwide, with knee OA being the most common in terms of prevalence (6% of adults > 30 years of age) (Zhang and Jordan, 2008). It is widely reported that knee OA is associated with compromised gait function in that it exacerbates the altered spatiotemporal gait parameters caused by aging, such as reduced stride length and speed and increased stride time, stance phase duration and double support time (Al-Zahrani and Bakheit, 2002; Astephen et al., 2008; Mills et al., 2013a). In addition to changes in mean spatiotemporal gait parameters, other studies have shown that knee OA is associated with changes in the variability of these parameters and the basic movement patterns that comprise the gait cycle (Kiss, 2011; Tochigi et al., 2012).

Research that has investigated the variability of one or more aspects of the gait cycle defines the area of study known as gait variability. Numerous studies have shown that gait variability is particularly sensitive to differences between healthy individuals and those with mild to moderate gait impairments. Evidence suggests that too much or too little variability is disadvantageous to the stability of the system, as has been shown by studies that have found both increased and decreased gait variability in different populations (Hausdorff, 2009; Tochigi et al., 2012). Studies on gait variability have largely investigated the regulation and timing of the gait pattern in various pathological conditions, including Parkinson's disease (Hausdorff, 2009), multiple sclerosis (Kalron, 2016), Alzheimer's disease (Wittwer et al., 2013), ALS (Hausdorff et al., 2000) and the changes in gait associated with healthy aging (Hausdorff et al., 1997; Kobsar et al., 2014a). A variety of different methods (cameras, pressure-sensitive mats and accelerometry) have been used to investigate a range of parameters that typically include linear measures such as the standard deviation (SD) or coefficient of variation of mean spatiotemporal gait parameters (e.g., stride time SD), or more sophisticated non-linear methods that aim to quantify the complexity of the gait cycle across different time spans (e.g., sample entropy, fractal scaling index) (Hausdorff et al., 1997; Tochigi et al., 2012; Kobsar et al., 2014a; Alkjaer et al., 2015). The application of accelerometry for gait variability analysis is advantageous because it allows for the collection of large amounts of data (i.e., potentially thousands of gait cycles) using unobtrusive sensors under natural walking conditions (Kobsar et al., 2014a). Conducting a similar analysis using a lab-based 3D camera system (for example) requires the use of a treadmill, which potentially alters the participant's internal rhythm (and consequently their gait variability) due to the external pacing imposed by the constant speed of the belt. It also provides the opportunity to take advantage of advanced analytical methods such as autocorrelation analysis (Moe-Nilssen and Helbostad, 2004), which can be used to extract discrete parameters such as the stride time and step time, in addition to using the entire acceleration waveform to determine the regularity (consistency of the stride-to-stride or step-to-step pattern) and symmetry (difference between step and stride regularity) of the gait cycle (Moe-Nilssen and Helbostad, 2004; Kobayashi et al., 2014; Kobsar et al., 2014a).

In general, studies that have investigated gait variability have produced favorable results that have advanced the understanding of the effect of different pathologies on gait function; however, the majority of these studies have focused on the effects of neural pathology (Moon et al., 2016) as opposed to diseases that directly affect the musculoskeletal system such as hip and knee OA. Several studies have found differences in select measures of gait variability (Yakhdani et al., 2010; Kiss, 2011; Tochigi et al., 2012; Gustafson et al., 2015) and gait symmetry (Mills et al., 2013b) in patients with unilateral and bilateral knee OA, respectively, presumably because pathophysiologically-induced changes to the morphology of the joint (e.g., decreased joint space, decreased range of motion, and increased pain) produce altered gait patterns as a result of CNS-mediated compensation strategies that develop over time to avoid pain, re-distribute joint loads and/or maintain dynamic stability under adverse conditions. Other studies have shown that sex and laterality (i.e., unilateral vs. bilateral incidence) are important factors that should be considered when investigating step regularity and gait symmetry in older adults with and without knee OA. Recently, Kobayashi et al. (2014) found differences in step regularity between older adult males and females, while Kiss (2011) found differences in cadence and step length variability between males and females with knee OA. With respect to gait symmetry, Mills et al. (2013b) have shown that between-limb kinematic asymmetries are greater for individuals with bilateral knee OA as opposed to unilateral knee OA. Consequently, the purpose of this study was to use an accelerometry-based unbiased autocorrelation procedure to determine the stride regularity, step regularity and gait symmetry of individuals with bilateral knee OA, and to compare these values to a group of age and sex-matched pain-free controls. It was hypothesized that males and females with knee OA would display less step and stride regularity than control participants and that their gait would be less symmetric. Based on the results of Kobayashi et al. (2014), it was also hypothesized that male participants in both groups would have lower step regularity than females.

## Methods

### Participants

Fifteen adults, 55 years of age or older (8 females, 7 males; 64.7 ± 6.8 years), with bilateral knee OA participated in the study. In addition, fifteen age and sex-matched older adults (8 females, 7 males; 66.1 ± 10.0 years) who presented with no knee pain or diagnosis of knee OA were recruited as control participants. Table 1 provides further information concerning the participant demographics. Inclusion criteria for the knee OA group consisted of having received a medical diagnosis of knee OA in addition to being able to walk comfortably for at least 9 min without the use of an assistive device (e.g., cane or walker). The severity of knee OA (as determined by the Kellgren-Lawrence grading scale) varied across participants, in that five participants had a K-L grade of 4 (in the most severe knee), eight had a grade of 3, and two had a grade of 2. Participants in the control group were selected using the same criteria as those for the knee OA group except for the presence of knee OA. Participants in either group were excluded if they had any recent surgery that affected their legs or lumbar spine, if they possessed OA in any other lower extremity joint (e.g., hip), if they had any neuromuscular disorders (e.g., Parkinson's disease, multiple sclerosis, etc.), history of stroke, cardiovascular disease, or any other medical condition or physical impairment that would affect their gait, balance, and/or their ability to walk at a steady pace for 10 min. This study was carried out in accordance with the recommendations of the University of Regina Research Ethics Board (REB) with written informed consent from all subjects. All subjects gave written informed consent in accordance with the Declaration of Helsinki. The protocol was approved by the University of Regina Research Ethics Board (REB-71S112).

**Table 1:** **Demographic and temporal gait parameter data for knee OA and control group participants**.

**Parameter**	**Control (Mean ±*SD*)**			**Knee OA (Mean ±*SD*)**		
	**Female (*n* = 8)**	**Male (*n* = 7)**	**Total (*n* = 15)**	**Female (*n* = 8)**	**Male (*n* = 7)**	**Total (*n* = 15)**
Age (years)	66.8±10.5	65.3±10.2	66.1±10.0	63.9±8.7	65.6±4.0	64.7±6.8
Height (cm)	**159.3 ± 4.0^†^**	**176.8 ± 8.5^†^**	167.5±11.0	**160.7 ± 7.6^†^**	**174.6 ± 5.4^†^**	167.2±9.7
Mass (kg)	**61.9 ± 9.5^†^**	**84.3 ± 16.2^†^**	72.4 ± 17.1^*^	**78.5 ± 10.0^†^**	**92.7 ± 6.6^†^**	85.2 ± 11.0^*^
BMI (kg/m^2^)	24.4±3.9	26.8±3.4	25.5 ± 3.8^*^	30.5±4.4	30.6±3.8	30.6 ± 4.0^*^
Stride time (ms)	979±74	1029±73	1002±76	1055±76	1046±73	1051±72
Step time (ms)	489±37	515±37	501±38	527±38	523±37	525±36

* *Results of the two-way ANOVA (group × sex) indicating significant differences between groups (p < 0.01) in bold*.

† *Significant differences between sexes (p < 0.01) are marked in bold*.

### Apparatus and procedure

The height and weight of each participant was recorded for the purpose of calculating the body mass index (BMI). The test procedure required the participants to walk around an oval 200-m indoor track at a consistent, self-selected speed for a period of 9 min. Self-selected speeds were chosen because they most accurately represent the natural gait pattern according to each participant's stature and other physical factors such as strength and flexibility (Clermont and Barden, 2016). A single triaxial accelerometer (GENEActiv, Cambridgeshire, UK) was attached to a belt that was located firmly at the lower back (L3) to approximate the total body center of mass (Moe-Nilssen and Helbostad, 2004; Kobsar et al., 2014b). For a depiction of the experimental setup please refer to Kobsar et al. (2014b). The accelerometer recorded continuous acceleration at a sampling rate of 100 Hz during the 9-min walking trial, which is consistent with sampling frequencies used by previous studies to determine measures of gait variability (Hartmann et al., 2009; Kobsar et al., 2014a).

### Data analysis

The data sets from the 9-min walking trials were reduced to 6 min by removing the first and last 90 s of each trial. The average number of steps for 6 min of walking was 682.1 (SD ± 45.1) for knee OA participants and 710.1 (SD ± 36.5) for control participants. This was done to ensure that the participants had sufficient time to achieve a steady-state walking speed prior to the analysis and to remove any potential gait irregularities associated with anticipating the termination of the trial (Lindemann et al., 2008). All data processing and gait parameter calculations were done using MATLAB version R2016A (The MathWorks Inc., Natick, MA). The signals from all three accelerometer axes were initially processed using a zero-lag, 4th order Butterworth low-pass filter with a cut-off frequency of 10 Hz. Subsequently, a negative peak-detection (local minima) method on the anteroposterior axis was used to identify each individual step to determine the step times for the 6-min time series (Terrier and Dériaz, 2011). From this series of step times, the mean step time and stride time were determined. Mean step time was defined as the average of all step times (i.e., right and left combined) and stride time was obtained by combining each set of subsequent left and right steps together. As per previous studies, a median filter was used to remove any potential outliers (defined as step times greater than three SDs from the median) in each subject's step time series (Hausdorff and Edelberg, 1997; Kobsar et al., 2014a).

**Figure 1:**
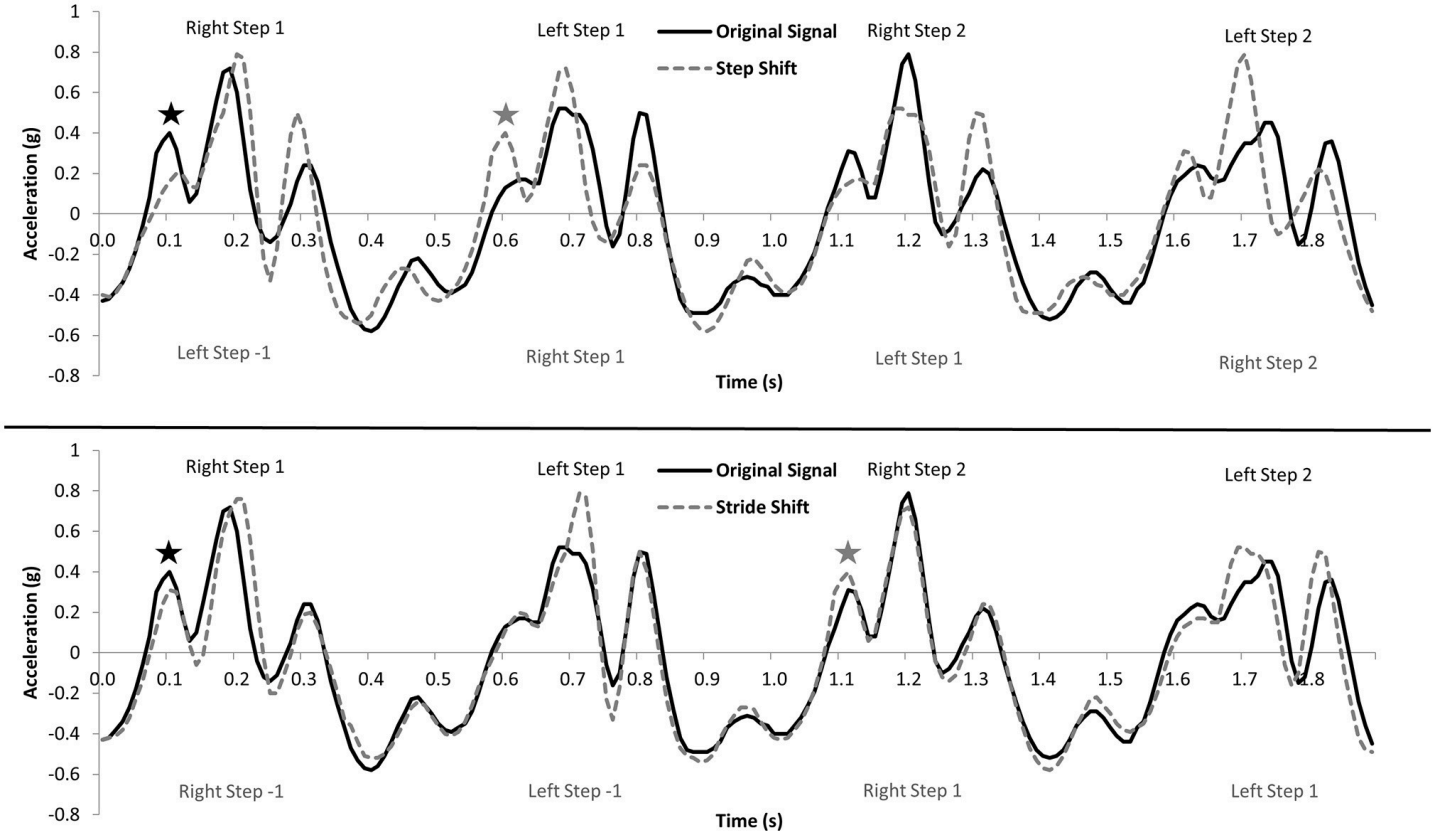
**Visual representation of the autocorrelation procedure for a series of four steps**. The top panel shows the correlation between the original signal and the signal shifted equivalent to the average step time, which defines step regularity. The bottom panel shows the correlation between the original signal and the signal shifted equivalent to the average stride time, which defines stride regularity. The ⋆ shows the original and step/stride shifted waveforms in the top and bottom panels, respectively. Adapted from Kobsar et al. (2014a), with permission from Elsevier.

To determine stride and step regularity, an unbiased autocorrelation procedure (see Figure 1) was used to measure the correlation of the acceleration signal for each step (first dominant period) or stride (second dominant period) at different periods of time (i.e., phase shifts) across each of the three accelerometer axes (Moe-Nilssen and Helbostad, 2004; Kobsar et al., 2014a). Step regularity was defined as the correlation between the original acceleration signal and the acceleration signal phase shifted to the average step time, whereas stride regularity was shifted to the average stride time (Kobsar et al., 2014a). These phase shifts were consistent with the first and second dominant periods of the unbiased autocorrelation coefficient, respectively (Moe-Nilssen and Helbostad, 2004; Kobayashi et al., 2014; Kobsar et al., 2014a). Gait symmetry (*Sym*) was defined as the percent difference between the regularity of steps (*StpReg*) and the regularity of strides (*StrReg*) for each of the three axes, with zero being perfect symmetry and larger values depicting greater levels of asymmetry (the difference between the consistency of strides and the consistency of steps) in the accelerometer waveform (Kobsar et al., 2014a).

$$Symmetry=\left\{\begin{array}{c} \underline{\left|StpReg\;-\;StrReg\right|}\\ (StpReg\;+\;StrReg)/2 \end{array}\right\}*100$$

Consequently, the accelerometer waveforms in the vertical (V), anteroposterior (AP), and mediolateral (ML) directions were phase-shifted using the autocorrelation procedure to determine the step regularity (*StpV, StpAP, StpML*), stride regularity (*StrV, StrAP, StrML*) and symmetry (*SymV, SymAP, SymML*) of knee OA and control group participants.

### Statistical analysis

Separate two-way (group × sex) ANOVAs were used to compare dependent variables for participant demographics (age, height, mass, and BMI), temporal gait parameters (stride time and step time) as well as stride regularity (*StrRegV, StrRegAP, StrRegML*), step regularity (*StpRegV*,*StpRegAP, StpRegML*) and gait symmetry (*SymV, SymAP, SymML*). *Post-hoc* comparisons using Fisher's LSD test were used to further identify any significant interaction effects. Effect sizes (partial η^2^) were determined for all comparisons according to the following categories: small (<0.06), moderate (0.06–0.14) and large (>0.14) (Cohen, 1988). All statistical analyses were performed using IBM SPSS Statistics 19.0 (SPSS Inc., Armonk, NY) with the significance level set to *p* < 0.05.

## Results

Mean values for age, height, mass, and BMI along with the temporal gait variables (step and stride time) for both groups are presented in Table 1.

Significant between group differences were found for mass [*F*_(1, 26)_ = 9.65, *p* = 0.005, η_*p*_^2^ = 0.271], and BMI [*F*_(1, 26)_ = 12.01, *p* = 0.002, η_*p*_^2^ = 0.317], indicating that participants with knee OA had a higher mass and a greater BMI than control group participants. Significant differences were also found between males and females of both groups for height [*F*_(1, 26)_ = 43.00, *p* = 0.000, η_*p*_^2^ = 0.623] and mass[*F*_(1, 26)_ = 20.61, *p* = 0.000, η_*p*_^2^ = 0.442], demonstrating that male participants were heavier and taller than their female counterparts. Mean differences between groups for stride time [*F*_(1, 26)_ = 2.91, *p* =.100, η_*p*_^2^ = 0.101] and step time [*F*_(1, 26)_ = 2.91, *p* = 0.100, η_*p*_^2^ = 0.101] approached significance with moderate effect sizes, suggesting that the strides and steps of knee OA participants took longer, which is indicative of slower walking speeds.

**Figure 2:**
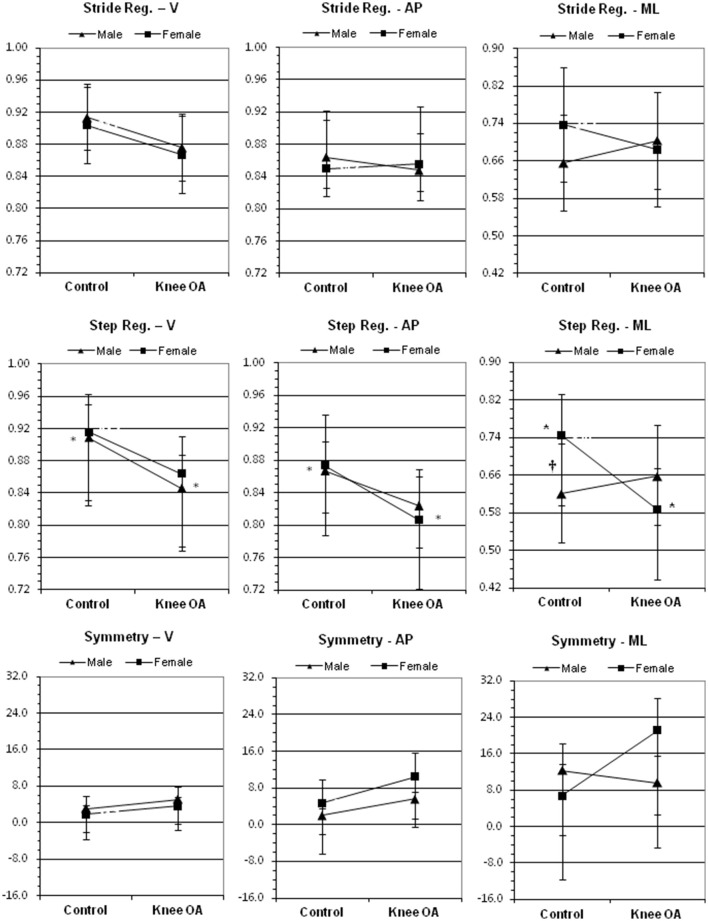
**Effects of group (control vs. knee OA) and sex (male vs. female) on stride regularity (top), step regularity (middle) and gait symmetry (bottom) in the vertical (V), anteroposterior (AP) and mediolateral (ML) directions**. The ^*^ indicates a significant difference (*p* < 0.05) between groups whereas the ^†^indicates a significant difference (*p* < 0.05) between male and female controls. Error bars = ± 1 standard deviation of the mean.

The two-way ANOVA results for stride regularity, step regularity and gait symmetry are summarized in Table 2. The mean values for stride regularity, step regularity and gait symmetry for participants in both groups (for each axis) are presented in Figure 2. Significant group main effects were found for step regularity in the vertical [*F*_(1, 26)_ = 5.28, *p* = 0.030, η_*p*_^2^ = 0.169] and anteroposterior directions [*F*_(1, 26)_ = 5.77, *p* = 0.024, η_*p*_^2^ = 0.182], indicating that the steps of both males and females with knee OA were completed with less regularity (i.e., were associated with less similar waveforms) than control participants. No significant main or interaction effects were found for stride regularity or gait symmetry in any of the three directions. A significant interaction effect [*F*_(1, 26)_ = 5.50, *p* = 0.027, η_*p*_^2^ = 0.175] was also found for step regularity in the mediolateral direction. *Post-hoc* comparisons revealed that mediolateral step regularity was significantly higher for female controls than for females with knee OA (*p* < 0.05), whereas the difference between males was non-significant. Further, mediolateral step regularity was significantly higher for female controls than for males (*p* < 0.05), whereas the difference between males and females in the knee OA group was not significant. All significant differences were associated with large effect sizes (η_*p*_^2^ > 0.14). Table 2 also shows that several additional comparisons (specifically, group *StrRegV*, group *SymAP*, and group^*^sex *SymML*) were associated with moderate effect sizes (η_*p*_^2^ = 0.06–0.14) and *p*-values that approached significance (*p* < 0.06, *p* < 0.07, and *p* < 0.08, respectively).

**Table 2:** **ANOVA results for stride regularity, step regularity and gait symmetry based on accelerometer axis (i.e., direction)**.

**Factor**	**Stride regularity**						**Step regularity**						**Gait symmetry**					
	**V**		**AP**		**ML**		**V**		**AP**		**ML**		**V**		**AP**		**ML**	
	***p***	**η_*p*_^2^**	***p***	**η_*p*_^2^**	***p***	**η_*p*_^2^**	***p***	**η_*p*_^2^**	***p***	**η_*p*_^2^**	***p***	**η_*p*_^2^**	***p***	**η_*p*_^2^**	***p***	**η_*p*_^2^**	***p***	**η_*p*_^2^**
Group	0.06	**0.13^†^**	0.79	0.00	0.93	0.00	0.03^*^	0.17	0.02^*^	0.18	0.17	0.07	0.22	0.06	0.07	**0.12^†^**	0.22	0.06
Sex	0.62	0.01	0.86	0.00	0.46	0.02	0.60	0.01	0.79	0.00	0.52	0.02	0.38	0.03	0.14	0.08	0.52	0.02
Group^*^Sex	0.99	0.00	0.57	0.01	0.24	0.05	0.83	0.00	0.60	0.01	0.03^*^	0.18	0.96	0.00	0.65	0.01	0.08	**0.12^†^**

*V, vertical; AP, anteroposterior; ML, mediolateral*.

* *Significant differences (p < 0.05) are indicated in bold. Effect sizes (partial η2) were determined for all comparisons according to the following categories: small (<0.06), moderate (0.06–0.14) and large (>0.14) (Hartmann et al., 2009)*.

† *Differences approaching significance with moderate effect sizes are indicated in bold*.

## Discussion

The purpose of this study was to compare the stride regularity, step regularity and gait symmetry of individuals with knee OA to age and sex-matched control participants, using an unbiased autocorrelation procedure based on data obtained from a triaxial accelerometer. It was hypothesized that males and females with knee OA would display less step and stride regularity than control participants and that their gait would be less symmetric. It was also hypothesized that male participants in both groups would have lower step regularity than females. Participants with knee OA had a higher mass and a greater BMI than control group participants (*p* < 0.01), which is consistent with the findings of other studies on knee OA (Landry et al., 2007). More importantly, the hypothesis with respect to step regularity was supported in that significant differences were found between knee OA and control group participants in the vertical and anteroposterior directions. Significant differences were also found for mediolateral step regularity between females with and without knee OA, and between males and females in the control group. To the best of our knowledge, this is the first study to have investigated stride and step regularity in individuals with knee OA using accelerometry-based autocorrelation analysis. Two other studies have investigated step and stride regularity in healthy older adults and the current findings are consistent with those results (Kobayashi et al., 2014; Kobsar et al., 2014a).

This study found significant differences in vertical and anteroposterior step regularity between participants with and without knee OA. This finding demonstrates that the similarity of step-to-step trunk acceleration waveforms was less regular (more variable) for individuals with knee OA compared to similarly-aged older adults with healthy knees. Only a limited number of studies have investigated gait variability in knee OA, and the findings of this study are generally consistent (in terms of increased variability) with the study by Kiss (2011) who found that knee OA participants demonstrated greater variability in step length, stance time, cadence, and double-support time than controls. However, these findings conflict with the results of Tochigi et al. (2012) and Yakhdani et al. (2010), who found that participants with knee OA possessed reduced variability of leg and knee motion compared to controls. In relation to this difference, it is important to note that Tochigi et al. (2012) and Yakhdani et al. (2010) not only used different methods (Sample Entropy and Lyapunov exponents, respectively), but more importantly quantified the variability of the affected limb as opposed to the variability of the approximate total body center of mass as was the case in this study. From a dynamical systems perspective, the variability of an injured limb may be inherently different from that of the center of mass during gait (Hamill et al., 2012). It is also important to note that significant differences in mass and BMI were found between knee OA and control group participants. To our knowledge, no studies have investigated the effect of body mass on step and stride regularity in older adults (or in younger adults), and as such it is not possible to know whether this had any effect on the results. Given that body mass and BMI are both important risk factors for knee OA (and are therefore common in this population), it is important that future studies take these factors into account. Further research is also needed to investigate the effect of knee OA on joint specific (knee) and total body variability to provide further insights into gait coordination and stability in this population. Additionally, a study by Lewek et al. (2006) found no difference in knee motion variability between unilateral knee OA participants and controls, but an increase in variability of the unaffected limb within the knee OA group. These mixed results demonstrate that additional research is needed to determine the relationship between knee OA and the various measures of gait variability.

With respect to the current findings, it is important to interpret the differences in anteroposterior step regularity in combination with the differences in stride regularity (Moe-Nilssen and Helbostad, 2004). Specifically, anteroposterior stride regularity was essentially the same for both groups, whereas step regularity was significantly less for the knee OA group. Low values for step regularity in combination with similarly low values for stride regularity indicate low regularity for both steps and strides, whereas low step regularity with a higher stride regularity indicates better regularity for strides than steps, which suggests bilateral asymmetry between left and right steps (Moe-Nilssen and Helbostad, 2004). This difference between step and stride regularity is also reflected in the gait symmetry measure, which in the case of the anteroposterior direction was associated with a moderate effect size (η_*p*_^2^ = 0.12) that approached significance (*p* < 0.07). Together these findings suggest that individuals with knee OA possessed greater bilateral step asymmetry than individuals with healthy knees. This indicates that the variability of the anteroposterior trunk acceleration pattern on a step-to-step basis was greater than normal in individuals with knee OA. It is possible that this may have occurred because of differences in knee OA severity between the two limbs, resulting in a less consistent pattern of motion between right and left steps. In relation to this point, it should be noted that the participants in this study possessed bilateral knee OA, and while it might seem reasonable to speculate that a similar study on unilateral knee OA participants would find an even more pronounced difference in anteroposterior step regularity, a recent study by Mills et al. (2013b) demonstrated that bilateral joint kinematic asymmetries were more prevalent in individuals with bilateral knee OA than unilateral knee OA. Mills et al. suggested that this may occur because individuals with unilateral disease maintain kinematic symmetry further into the knee OA process than those with bilateral disease (Mills et al., 2013b). Concerning the importance of the anteroposterior direction, it has also been suggested that differences in anteroposterior stride and step regularity may reflect differences in the control of the propulsion and braking phases of gait (Kobsar et al., 2014a), whereas Moe-Nilssen et al. (2010) found that anteroposterior step autocorrelation (i.e., anteroposterior step regularity) was highly associated (negatively correlated) with step length variability. Consequently, in addition to bilateral asymmetry, it is possible that reduced anteroposterior step regularity indicates a diminished capacity to control step length in individuals with bilateral knee OA.

With respect to the difference in vertical step regularity, it is important to note that the vertical stride regularity of knee OA participants was also lower than that of the control group (which approached significance at *p* < 0.06 with a moderate effect size of η_*p*_^2^ = 0.13), suggesting that a more general lack of regularity for both steps and strides existed in this direction for those with knee OA. This is supported by the fact that the gait symmetry values for the knee OA group were lower for the vertical direction (indicating greater symmetry between steps and strides) than for the anteroposterior direction. Consequently, the findings for the vertical direction suggest a more general lack of consistency in the overall pattern of gait for individuals with knee OA. In terms of what these results might signify, it is conceivable that they could reflect inconsistencies in loading and/or differences in step time variability. Evidence exists to suggest the latter as Moe-Nilssen et al. (2010) have shown that vertical step autocorrelation (i.e., step regularity) is highly related to step time variability. Therefore, in addition to greater bilateral step asymmetry, it is conceivable that the differences in anteroposterior and vertical step regularity together indicate that individuals with knee OA possess greater step length and step time variability than participants with healthy knees. If supported by future research, these findings suggest that waist-mounted accelerometry and autocorrelation analysis could be used to conduct clinical assessments of gait variability in individuals with knee OA using a simple, non-invasive, self-paced walking test. Such assessments have the potential to evaluate differences in gait between individuals with different levels of knee OA severity, to evaluate individuals before and after knee replacement surgery and to evaluate the functional outcomes of post-surgical physical therapy. Given the advantages of using accelerometers to determine measures of gait variability (i.e., small size, low cost, collection of large amounts of data and easily placed on the body in multiple locations, not to mention their ubiquity in most modern smart phones), the potential for clinical application is substantial. This potential will be realized if future studies can demonstrate that gait variability parameters, such as step and stride regularity, have practical value as clinical outcome measures that can be applied to the assessment and treatment of knee OA.

This study also found a significant interaction effect for mediolateral step regularity, such that females with knee OA had significantly lower mediolateral step regularity than female controls, while females within the control group had significantly higher mediolateral step regularity than males. These findings were surprising because it was not hypothesized that group differences in step regularity, stride regularity or gait symmetry would be dependent on sex. For control participants, the data clearly shows that an important difference exists between males and females for both mediolateral step and stride regularity, such that males had lower step and stride regularity than females. This is in contrast to the vertical and anteroposterior directions in which the mean values were quite similar. It is interesting to note that a recent study by Kobayashi et al. (2014), which investigated step and stride regularity in young and older adults, found differences in step regularity for the vertical and anteroposterior directions between males and females in the older adult group, but not for the young group. Unfortunately, Kobayashi et al. (2014) did not provide data for the mediolateral direction, but the findings for step regularity are consistent with the current study in terms of older adult males displaying lower step regularity than females, albeit for different directions. This study did not find any differences in vertical and anteroposterior step regularity between older adult male and female controls as Kobayashi et al. (2014) did; however, it is possible that this could be the result of the small number of steps that were used to determine step and stride regularity in the Kobayashi et al. (2014) study (participants walked the length of a 7 m walkway twice, which likely amounted to approximately 20–30 steps, as opposed to the approximately 700 steps used in this study).

Given that Moe-Nilssen et al. (2010) found no relationship between mediolateral step regularity and other gait variability parameters such as step time and step length variability, it is possible that mediolateral trunk variability may serve a different purpose in locomotor control compared to the other two directions. This is consistent with the observations made by Kobsar et al. (2014a), who suggested that mediolateral step regularity may be indicative of dynamic balance control. Regardless of what the difference signifies, to our knowledge this is the first study to have found a significant difference in mediolateral step regularity between older adult males and females and between females with and without knee OA. If this parameter is representative of dynamic balance control, it suggests that older adult females have better postural stability in the mediolateral direction than men. This may occur because men on average tend to be taller than women, and in theory maintaining dynamic stability with an aging neuromuscular control system should be more challenging for those individuals with a higher center of mass. However, it is also interesting that the relationship between male and female mediolateral step regularity appears to be negated in the presence of knee OA. Given that no previous research exists on step or stride regularity in knee OA it is difficult to speculate about what the cause of this might be. Nevertheless, it can be inferred that knee OA appears to affect the consistency of mediolateral trunk acceleration patterns more for women than for men, suggesting that women with knee OA may experience a greater loss of dynamic balance control than men. It is also conceivable that mediolateral step regularity might be associated with fall risk and/or the etiology of knee OA (given that knee OA is more common in women than in men), and as such this is an important finding that should be investigated further, both in healthy older adults and in adults with knee OA.

While the main strength of this study is the use of an accelerometer-based approach to investigate step/stride regularity and gait symmetry in older adults with knee OA, there were several limitations that should be considered. First, the participants were not matched according to body mass or BMI, and given that significant differences existed between the groups for these parameters, it is conceivable that these differences may have affected the results. Second, the sample size was relatively small and limited to participants with bilateral knee OA. The sample was also heterogeneous with respect to knee OA severity (i.e., K-L grade range of 2–4), both between limbs and between participants, and as such these factors may have also affected the results. Given that this study provides preliminary evidence to suggest a relationship between knee OA and step regularity, future studies should attempt to stratify participants according to severity.

## Conclusion

This study found that males and females with knee OA possessed significantly less vertical and anteroposterior step regularity than similarly-aged control group participants with pain-free knees. The findings suggest that the presence of knee OA affects the control of gait such that the consistency of the step pattern (more than the stride pattern) is compromised. Based on these results, it is not possible to know whether this is a direct consequence of the effects of knee OA itself (for e.g., pain and reduced range of motion), or if the increased variability is reflective of one or more compensation strategies employed by the CNS to manage the pathomechanics caused by the structural deficiencies of the joint. The study also found that mediolateral step regularity was lower for females with knee OA and lower for males than for females in the control group, suggesting that the relationship between mediolateral step regularity and knee OA is different in males and females. To our knowledge this is the first study to investigate stride regularity, step regularity and gait symmetry in individuals with knee OA, and the findings suggest that future research is needed to determine the specific aspects of gait control that are represented by these parameters (for e.g., is there a relationship between mediolateral step regularity and step width variability or dynamic balance control?). Given that this study found differences in step regularity with a relatively small number of bilateral knee OA participants, future studies (with larger sample sizes) should investigate unilateral knee OA and the effect of knee OA severity on step and stride regularity. The application of accelerometry-based gait regularity analysis for the assessment and treatment of knee OA in the clinical setting should also be investigated.

## Author contributions

JB authored the manuscript and was involved with all aspects of the study, CC was involved with the data collection and analysis, DK conducted the data analysis and OB assisted with manuscript preparation and revision.

### Conflict of interest statement

The authors declare that the research was conducted in the absence of any commercial or financial relationships that could be construed as a potential conflict of interest.
